# Looking for the Best Way to Come Off Steroids Safely? Exploring Post‐Cycle Therapy, Cessation, and Recovery Discourse and Practice in Australian Steroid Consumer Forums

**DOI:** 10.1111/dar.70042

**Published:** 2025-10-01

**Authors:** Jonathan Easton, Bonnie Grant, Geoffrey Spurling, Raphael Magnolini, Timothy Piatkowski

**Affiliations:** ^1^ School of Applied Psychology Griffith University Brisbane Australia; ^2^ Section of Investigative Medicine, Commonwealth Building Imperial College London, Hammersmith Hospital London UK; ^3^ General Practice Clinical Unit, School of Clinical Medicine Royal Brisbane & Women's Hospital, University of Queensland Brisbane Australia; ^4^ Southern Queensland Centre of Excellence in Aboriginal and Torres Strait Islander Primary Health Care Metro South Health Brisbane Australia; ^5^ Arud Centre for Addiction Medicine Zurich Switzerland; ^6^ Institute of Primary Care, University Hospital Zurich University of Zurich Zurich Switzerland; ^7^ Griffith Centre for Mental Health Griffith University Brisbane Australia; ^8^ Queensland Injectors Voice for Advocacy and Action Sunshine Coast Australia; ^9^ Centre for Health Services Research The University of Queensland Brisbane Australia

**Keywords:** anabolic‐androgenic steroids, cessation, harm reduction, post‐cycle therapy, recovery

## Abstract

**Introduction:**

Anabolic‐androgenic steroid (AAS) use is rising in Australia; however, discontinuation poses challenges due to withdrawal symptoms and a lack of clinical guidance. Therefore, the aim of this study was to explore online discourses around post‐cycle therapy (PCT), including perceived health risks, benefits and barriers, among people who use AAS.

**Methods:**

This study employed a digital ethnographic approach to explore Australian ‘steroid forums’ discussing PCT. Data were collected by extracting posts containing relevant keywords across four selected forums, with analysis guided by the Health Belief Model to identify barriers, facilitators and perceptions related to PCT.

**Results:**

A total of 150 threads and 5059 posts from 580 unique forum users were analysed. Key barriers to PCT engagement included social influences, aggressive anti‐PCT discourse and regulatory constraints. Benefits of PCT were recognised in preventing side effects and preserving muscle gains. Consumers shared concerns about hormonal imbalance, sexual health and long‐term damage, which were driving forces behind seeking advice on PCT.

**Discussion and Conclusions:**

This study highlights the role of online forums in shaping AAS consumers' understanding of PCT, where peer‐driven knowledge networks facilitate harm reduction. However, inconsistent advice and the lack of clear clinical guidelines, compounded by the lack of prospective studies, contribute to uncertainty and risk. This underscores the need for evidence‐based, non‐stigmatising support in healthcare.

## 1 Introduction

Anabolic‐androgenic steroids (AAS) are synthetic compounds that enhance muscle growth and performance by mimicking testosterone [1]. While AAS use was historically linked to competitive athletes, recent evidence shows that most users seek improved aesthetic appearance [2, 3]. Motivations include increasing muscle mass, reducing body fat, and enhancing recovery from training and injuries [4, 5]. In Australia, AAS use is rising, with new initiates to AAS use (injecting for less than three years) increasing from 18% in 2019 to 26% in 2023 [6]. Although AAS use is increasingly common, discontinuation poses significant challenges, as consumers often experience withdrawal symptoms and long‐term hormonal disruptions [7]. However, they face a lack of clinical guidance on effective cessation and recovery strategies, including post‐cycle therapy (PCT), an unproven regime used by men to restore endogenous testosterone production and mitigate withdrawal effects [8]. In the absence of standardised medical protocols, AAS consumers rely on peer networks and online forums to navigate all aspects of use [9, 10], including harm reduction, dosing, cycle planning, PCT and managing side effects. This study sought to explore online discourses around PCT, including perceived health risks, benefits and barriers among people who use AAS.

Upon discontinuation of AAS, people frequently experience withdrawal symptoms that can be categorised into acute and chronic phases. Acute symptoms, occurring within the first few days of cessation, include headaches, tremors, palpitations and nausea [7]. As the acute phase subsides, chronic symptoms emerge, most notably a transient hypogonadism [7]. Hypogonadism is particularly distressing due to its symptoms of erectile dysfunction, fatigue, persistent low libido and mood disorders [7, 11]. The severity of these symptoms can act as a significant barrier to AAS cessation, as individuals may be reluctant to discontinue use due to the physiological and psychological difficulties associated with hormonal disruption [12]. As a result, some men may go on to develop a dangerous AAS dependence syndrome [13]. These patterns of testosterone use and continuation are closely linked to cultural constructions of masculinity [14, 15], where physical strength, muscularity and sexual function are central to identity and social expectations among Australian men [16]. The distress caused by withdrawal symptoms among men is likely often experienced not only as a health issue but as a threat to masculine ideals, which can reinforce continuation or dependence on AAS [17]. In response to these challenges, many AAS consumers turn to pharmacological interventions aimed at restoring endogenous testosterone production and alleviating hypogonadal symptoms following cessation.

PCT is a widely adopted, yet informal and unproven, endocrine treatment approach and harm reduction strategy used by AAS consumers to mitigate withdrawal symptoms and accelerate recovery of the hypothalamic–pituitary‐gonadal axis after permanent cessation [18, 19]. PCT regimes vary significantly; however, they typically involve self‐administering a combination of pharmaceutical substances over a period of 2–12 weeks, including human chorionic gonadotropin (hCG), selective oestrogen receptor modulators and aromatase inhibitors [20, 21, 22]. While hCG directly stimulates testosterone production in the testes, selective oestrogen receptor modulators and aromatase inhibitors function by reducing estrogenic feedback, thereby promoting gonadotropin‐releasing hormone (GnRH) and pituitary gonadotropin secretion [23, 24, 25]. PCT is used by AAS consumers to preserve muscle mass gained during usage cycles [12]. However, concerns persist about the legitimacy and effectiveness of PCT substances, with some expressing doubts about product authenticity [26]. While recent studies have explored PCT practices [8, 27, 28], gaps remain in understanding who uses PCT, how information is shared and which populations benefit. Evidence on its effectiveness, safety, tolerability, optimal duration, dosing and combinations is limited, with a lack of robust prospective studies.

The absence of standardised medical protocols and clinical guidance for AAS cessation and recovery is compounded by a broader structural issue: the lack of knowledge and education among healthcare professionals regarding AAS use. Research consistently shows that healthcare providers are underprepared to meet AAS consumers' needs [29, 30, 31], creating barriers to accessing appropriate care. Consumers often feel displaced or misunderstood when seeking support for cessation or recovery [29, 32]. These challenges are compounded by broader dynamics of structural stigma, where drug use including AAS [33] remains highly marginalised within healthcare and policy settings [34]. This stigma not only limits service access but also delegitimises the knowledge and experiences of AAS consumers [33], further entrenching their reliance on non‐clinical sources of support. In response, many turn to peer networks, where personal experiences and insights are shared and valued [35, 36]. These communities, including online forums [37], are seen as more accessible and trustworthy than medical advice, providing crucial spaces for AAS consumers to navigate challenges and seek guidance on harm reduction strategies like PCT. In this sense, peer‐led engagement with AAS and PCT can be understood within the broader harm reduction movement [38], which seeks to prioritise safety, autonomy and dignity over abstinence‐based or punitive approaches.

For people who use AAS, this shift toward peer‐led knowledge leverages the concept of ‘ethnopharmacology’, also known as ‘bro‐science’, which represents communities' lived‐living experience of AAS as a legitimate way of understanding and using these substances [39, 40]. Drawing on previous scholars [35, 39], Piatkowski et al. use the term ‘ethnopharmacological connoisseurship’ to describe the situated, practice‐based knowledge that emerges when people who use AAS develop finely tuned understandings of drug sourcing, quality, dosing, and physiological effects through both embodied experience and peer exchange [41]. This concept extends beyond individual expertise, operating as a form of collective, culturally embedded knowledge that challenges biomedical dominance by recognising the value of community‐led harm reduction. In this way, AAS consumers should be viewed as active participants in developing strategies based on their unique understandings of the substances they use. Through situated and contextually relevant knowledge gained from their lived‐living expertise, they not only mitigate risks but are also able to contribute valuable insights to harm reduction and health promotion when appropriately engaged.

This reliance on peer‐led knowledge and ethnopharmacological expertise highlights the importance of understanding the factors that shape health‐related decision‐making within AAS communities. The Health Belief Model (HBM) offers a comprehensive theoretical framework for understanding how individuals perceive their health and the factors that motivate them to engage in health‐protective behaviours. The model suggests that health behaviours are shaped by a combination of personal perceptions and external influences. Central to the HBM are several key constructs: perceived susceptibility, perceived severity, perceived benefits, perceived barriers and cues to action [42]. Perceived susceptibility refers to an individual's belief about their risk of experiencing a particular health issue, while perceived severity reflects their perception of the seriousness of the potential consequences of that health issue. Perceived benefits encompass the individual's belief in the effectiveness of a health behaviour in reducing the risk or severity of the issue, while perceived barriers address the factors that may hinder the adoption of the behaviour. Cues to action are triggers that prompt individuals to take action, such as reminders or social influences. Together, these constructs provide a lens through which to understand how people make decisions about their health, including those related to engaging in harm reduction strategies such as PCT. In AAS communities, health behaviours are negotiated through dynamic interactions shaped by cultural norms, shared experiences and identity performances that extend beyond individual perceptions [26]. Although the HBM alone does not explicitly account for how these community dynamics influence the acceptance and interpretation of health information, it has been used in research as a foundational framework, recognising that health decisions are embedded within complex social environments and peer knowledge systems [43]. Therefore, the aim of this study was to explore online discourses around PCT, including perceived health risks, benefits and barriers, among people who use AAS.

## 2 Methods

### 2.1 Study Design and Ethics

This research was approved by the Griffith University Human Research Ethics Committee (Approval number: 2024/314). In line with the British Psychological Society's guidelines for internet‐mediated research [44], ethical issues such as respect for autonomy, privacy and dignity, as well as scientific integrity, social responsibility and harm minimisation, were carefully considered. Forum data were obtained unobtrusively from publicly accessible threads, with no interaction between researchers and participants. While posts often discussed potentially illicit activities, they were made in public spaces where forum users were aware their content could be read by anyone. We note extant research [45] shows the use of pre‐existing online data minimises participant burden, avoids influencing community dynamics and provides a naturalistic account of peer‐to‐peer discourse.

### 2.2 Data Collection

Data were collected through digital ethnography, involving extensive lurking on selected forums [46]. An online search for ‘Australian bodybuilding forums’ and ‘Australian steroid forums’ was conducted using Google.com.au. Forums were selected for analysis if they met the inclusion criteria of: (i) being predominantly Australian; (ii) having active users daily; and (iii) being accepting of and having a significant amount of discussion surrounding PCT. This was verified through the senior author's (TP) long‐standing involvement in the AAS community, including prior participation in some of these forums and ongoing engagement in Australian AAS consumer groups on encrypted messaging platforms (e.g., Telegram). Four forums initially fit the criterion: brotherhoodofpain.com, ausbb.com, www.evolutionary.org and ugltalk.is, but ugltalk.is was excluded from analysis as the forum changed platform structure at the beginning of data collection. It is known that while the selected forums are predominantly Australian, there are users from countries other than Australia. However, consumers outside Australia were expected to be a minority based on past research [10]. This study collected primary data from online forums by systematically extracting posts from threads containing the keywords ‘PCT’ or ‘Post‐cycle‐therapy’ in order of most to least recent. Using a manual data scraping approach, data from the first 10 pages or 50 threads, whichever came first depending on the structure of the forum, were collected. These cut‐offs were established at this amount due to the possible amount of information contained on these forums in relation to the topic [10]. Extracted threads and posts of these forums included usernames and were copied into textual management software for analysis, but usernames were excluded from any final quotes used in the final analysis.

### 2.3 Data Analysis

A netnographic [47, 48] approach was employed to analyse forum discussions, drawing on iterative categorisation [49] to code and then interpret [50] the digital interactions. Netnography was selected as it emphasises the social and cultural context in which posts are produced, enabling interpretation grounded in community practices, language and shared meanings rather than solely in textual form [47]. The HBM provided a deductive framework to explore key constructs such as perceived susceptibility to health risks, perceived severity of adverse outcomes, perceived benefits of PCT, barriers to engaging in PCT, cues to action and self‐efficacy in managing post‐cycle recovery. The research team's positionality combines lived‐living experience and clinical expertise. JE (undergraduate student and fitness enthusiast) conducted the research under TP's supervision. TP is a peer researcher with lived‐living experience and extensive, long‐term engagement in the AAS community, including prior participation in the analysed forums and ongoing involvement in Australian AAS consumer networks hosted on encrypted platforms (e.g., Telegram). TP also maintains contact with forum moderators and community leaders across multiple platforms, providing deep contextual insight into community norms and dynamics. Medical doctors and researchers with expertise in drugs (GS) and AAS (BG, RM) provided specialist knowledge on pharmacology, endocrinology and harm reduction practice. This interdisciplinary collaboration between clinicians and a peer researcher (TP) ensured that interpretations were both scientifically robust and grounded in the lived‐living experiences of AAS use, enabling a more comprehensive and contextually accurate analysis. Together, this positionality informed the analysis by allowing nuanced interpretation of terminology, humour, implicit meanings and the significance of certain exchanges that might otherwise be overlooked.

Data were imported into NVivo 12 (QSR) and analysed using iterative categorisation. Initially, all available data were coded to identify and develop themes related to PCT practices and perceptions. These preliminary codes were then subjected to multiple rounds of review and reclassification using the HBM as a guiding lens. Importantly, we recognise the limitations of the HBM when applied to peer‐led online knowledge systems, where health‐related decision‐making is not solely an individual cognitive process but also a socially negotiated one [26]. The iterative analytical process thus incorporated a reflexive engagement with the sociocultural and interactive dynamics of the forums, allowing us to interpret how collective meaning‐making and community norms intersect with individual health beliefs. For this reason, a critical realist perspective underpinned the analysis, acknowledging that while an objective social reality regarding PCT exists, our understanding is shaped by individual perceptions and contextual influences. This perspective recognises that while there are objective physiological effects and risks associated with PCT and AAS cessation, individuals' behaviours and decisions are profoundly shaped by socially mediated beliefs, cultural norms and identity [51]. This stance also reflected the interdisciplinary composition of the research team, combining clinical expertise with lived experience to capture both the material realities and the subjective meanings influencing PCT engagement. In practice, this meant that initially the data analysis incorporated ongoing collaborative discussions between JE and TP, grounded in reflexivity and a shared process of meaning‐making rather than consensus seeking. This iterative interpretive process was enriched through engagement with clinician–researchers (BG, RM, GS), whose perspectives were incorporated at multiple stages to refine and extend the analysis. As a result, the theme categories identified and developed from this process reveal both the barriers and facilitators influencing PCT engagement as well as the complex interplay between clinical and peer perspectives. These five theme categories are presented in the next section as a guiding framework for interpretation.

## 3 Results

In total, 150 threads and 5059 individual posts from 580 unique forum users were included in this analysis.

### 3.1 Perceived Barriers to Post‐Cycle Therapy

People who use AAS articulated a range of factors that discouraged engagement in health‐seeking or health‐promoting behaviours. Consumers highlighted social influences, such as peers' perceptions, knowledge and experiences, as shaping forum discussions in ways that could deter people from considering AAS cessation or engaging in PCT. A recurring point in their responses was the emotional and psychological impact of preparing for PCT or stopping AAS use. Many described feelings of uncertainty, anxiety or reluctance, which were reinforced through peer discussions.

You won't feel amazing, but you'll definitely feel a lot less shitty than if you didn't run a good PCT.

Getting ready to come off and I've always felt like shit even on super mild PCT.

This illustrates how shared experiences and community narratives form part of the information landscape that people engage with, which in some cases may coincide with lower prioritisation of formal health interventions, potentially creating barriers.

Forum discussions often reflected subjective perspectives of people with lived experience of AAS use, with some consumers appearing more focused on short‐term health improvements rather than long‐term health promotion or longevity.

I'm just focused on getting through this cycle without feeling like shit and worrying about that stuff [fertility discussion preceding] isn't an issue right now.

One key environmental barrier within these online AAS community spaces was the dominance of strong beliefs and opinions about PCT. These views were often expressed strongly, carrying negative connotations, not necessarily aligned with the aim of supporting less experienced consumers in adopting more effective PCT protocols, but rather in encouraging alternative approaches that prioritised staying on cycle over post‐cycle recovery. For example, some responses dismissed PCT altogether:

PCT drugs? I thought we were done with this shit.

These interactions illustrate how social norms and group cohesion can strongly influence peoples' health decisions. In this context, peer discourse often discouraged engagement with PCT, shaping attitudes and behaviours around recovery and long‐term health strategies.

Discussions around professional medical advice and government policy were less popular than other forum topics but still had a notable impact on consumers' ability to engage with PCT.

Doctors usually don't know what I'm talking about [when discussing AAS] or just tell me to stop using [AAS]. So I don't go [to the doctor] anymore.

Institutional barriers, including law enforcement and regulatory restrictions, influenced access and decision‐making:

Shitty news update. My PCT has been intercepted by Police. What are my chances of getting it through? Also, what would happen if I got busted again (after the previous letter already)? I hear it's three warnings before you're in serious trouble.

These responses highlight how policy and enforcement measures create additional obstacles for individuals seeking to manage their health. Criminalisation and regulatory constraints not only limit access to PCT but also contribute to marginalisation, which can hinder the development of community spaces where harm reduction strategies are discussed and adopted.

### 3.2 Perceived Benefits to Post‐Cycle Therapy

AAS consumers discussed differing views on the necessity of PCT, with some emphasising its role in mitigating side effects and speeding up recovery, while others suggested that the absence of symptoms indicated no need for intervention. Many consumers believed PCT was crucial for maintaining *gains* and minimising recovery time. Forum discussions reflected these contrasting opinions, shaping perceptions of the importance and urgency of post‐cycle interventions.

I have HCG [human chorionic gonadotropin], Clomid [clomiphene], Nolvadex [tamoxifen] on hand. Just looking for the best way to come off [AAS] safely with minimal sides. Thanks!

In contrast, other consumers emphasised the risks associated with neglecting PCT. As one participant warned:

Yes. If you come off and do not do a PCT, you run the risk of taking a long time to recover and losing a lot of your hard‐earned gains.

Beyond these individual statements, analysis also revealed how education and information sharing in these forums play a vital role in shaping consumer beliefs and expectations.

Love to see a PCT log. It is an extremely important part of any cycle and often gets overlooked.

I've included HCG [human chorionic gonadotropin], 5000iu in my PCT after using [brand name] 100mg/ml and it really helped restore my natural testosterone levels. The recovery has been smoother, and I didn't feel any of the usual fatigue after a cycle. [brand name's] HCG [human chorionic gonadotropin] was effective, I'm very pleased with it!

Education and information provision were perceived as important routes to improving health knowledge and efficacy of PCT protocols. These examples suggest that the forums serve as a dynamic space for peer education, where sharing logs, blood results and personal recovery experiences not only validates the benefits of PCT but also motivates others to adopt similar practices. However, not all interactions reinforce a positive view of PCT. Some posts introduce a cautionary tone, questioning delays or inconsistent adherence to protocols:

Why did you stop your previous log? That would have prevented you from making this mistake in the first place get it going as soon as possible.

These exchanges demonstrate how anonymous forum discussions enable consumers to reassess and refine PCT protocols. Anonymity fosters open sharing of logs, blood results and personal experiences, which reveal the benefits of structured PCT. Many participants emphasised that effective PCT is essential not only for mitigating side effects but also for preserving muscle mass and strength, which are key goals of AAS use.

### 3.3 Perceived Susceptibility of Non‐Engagement in Post‐Cycle Therapy

Many AAS consumers expressed significant concern about the potential adverse effects of AAS cycles. They frame these risks in terms of hormonal imbalances and long‐term health consequences, such as testicular atrophy and impaired fertility:

I'm worried about crashing and getting ‘tren dick’ [trenbolone dick] perhaps I should not use Arimidex [anastrozole] too often during cycle so as not to kill too much estrogen levels?

Another consumer raised specific concerns about reproductive health:

Just went for a sperm test because of personal reason and I have no sperm.

Would this be caused by the no PCT?

AAS consumers illustrated the anxiety surrounding the disruption of hormonal balance and the potential for severe health damage if PCT is not properly managed. Others cautioned against attempting PCT after prolonged cycles or self‐administered TRT, warning that recovery can be challenging:

You'll have a VERY hard time trying to do PCT after such a long cycle and self‐TRT [testosterone replacement therapy], maybe will take 6 months and will be painful and depressing, why do you want to do it?

A recurring theme in forum discussions is the concern over how a given AAS cycle may affect hormonal health, particularly testicular function and the risk of gynecomastia, which in turn drives many consumers to seek advice or initiate PCT out of fear of long‐term ill health. Some AAS consumers advocate a proactive approach, emphasising that adverse outcomes like testicular atrophy can be completely avoided with proper research and adherence to best practices.

Testicular atrophy (shrinkage) is completely avoidable if you do your research and aren't a tight arse.

Another consumer elaborated on the benefits of maintaining testicular function by stating,

By maintaining testicular function though, you eliminate (or at least massively reduce) the risk of ending up sterile or on HRT [hormone replacement therapy]. In my opinion, if you have the money to cycle, you have the money for HCG [human chorionic gonadotropin].

Here, the emphasis was on the idea that investing in proper recovery protocols, such as the use of HCG, is both a rational and necessary expense for those who cycle AAS. One user warned,

It could take years for your balls to recover without a PCT.

Conversely, some forum discussions reflected a dismissive attitude toward PCT, suggesting that in the absence of immediate negative symptoms, engaging in recovery protocols may not be necessary. For example:

PCT is retarded anyway, no matter what you take you run the risk of not bouncing back completely. If that's the way your headed I hate to say this but just stay on, shut yourself down and get on TRT [testosterone replacement therapy].

These contrasting views highlight a clear divergence: while some consumers emphasise the critical role of PCT in preventing long‐term damage and preserving ‘*gains*’, others, often drawing on their personal experience, underestimate these risks. For instance:

Last one I cut short because I figured I'm young enough my body will go back to normal without these drugs.

Collectively, these narratives show that while fear of negative health outcomes can motivate PCT engagement, responses vary. Some AAS consumers adopt proactive self‐care, while others, lacking immediate symptoms, underestimate risks and are unprepared for consequences.

### 3.4 Perceived Severity of Non‐Engagement in Post‐Cycle Therapy

Forum discussions revealed that AAS consumers often share both personal and observed experiences of adverse physical changes, such as increased oestrogen production leading to gynecomastia or other hormonal imbalances, which instilled a sense of urgency or fear. They reported concerns that failing to implement PCT will lead to significant long‐term damage, a view that can serve as an immediate trigger for behaviour change. For example, one user warns:

Without PCT, there'll be ridiculous estrogen produced and that leads to gynecomastia [breast tissue growth in males].

This comment underscores the anxiety over visible physical side effects, reinforcing the idea that proper PCT is essential to prevent undesirable outcomes. Beyond concerns about these effects, a significant motivation for seeking PCT advice stems from the desire to maintain the physical gains achieved during an AAS cycle. Many consumers stress that skipping PCT could jeopardise their *hard‐earned* progress:

PCT is vital to keep your gains, or you will lose them.

Further discussion in the forums also highlights debates over the appropriateness of different recovery drugs, such as Triptorelin, a peptide that stimulates the release of gonadotropin‐releasing hormone and is sometimes used in AAS recovery.

It's [Triptorelin] a scary substance because too much and you can chemically castrate yourself.

Forum members exchange advice based on personal experiences with successful and unsuccessful protocols, often reiterating information circulated within these spaces. Discussions frequently reference drugs used both adjacently to AAS (e.g., anastrazole) and as part of PCT (e.g., tamoxifen).

Im not upset with the original poster… I just hate Arimidex [anastrozole] & Nolvadex [tamoxifen]. They are blunt instruments and have terrible side effects that are like time bombs. The main one being loss of bone density.

AAS consumers expressed frustration with the use of multiple PCT drugs, particularly in the context of their perceived negative impacts on hormonal balance. One AAS consumer, reflecting the emotional intensity of the forum discussions, stated:

Who the FUCK told you to take all that shit? You feel like shit because you're taking a bunch of shit drugs, and your natural testosterone is shut down. Oh my fucking god I'm too pissed at the moment to begin typing a helpful response. Ok, for starters… explain in your own words why you are taking 40 fucking milligrams of Nolvadex [tamoxifen] a day?… or at all? Why are you ALSO taking Arimidex [anastrozole] at 1mg DAILY??? Congrats, you now have NO testosterone or estrogen. Go slap the fuck out of whomever told you to do this. Holy fuck.

Discussions such as this illustrates the concern among AAS consumers for each other. Particularly that such protocols may exacerbate health risks, especially in relation to hormonal dysregulation. The frustration expressed here reflects a broader concern in the community about the circulation of unverified, potentially harmful advice.

### 3.5 Cues to Action to Promote Post‐Cycle Therapy Engagement

AAS consumers often seek guidance when facing uncertainty about PCT protocols or experiencing unexpected health outcomes after AAS use. Forums may provide consumers with a support hub when they feel unwell, struggle with motivation, or experience hormonal imbalances post‐cycle. These concerns typically centre on whether their PCT was effective and how to manage lingering health effects. One consumer shared their experience:

I'm not feeling great at all don't have any motivation to work out my libido is down and I don't feel healthy in general I may have not done the best PCT can you recommend a supplement that I can start taking out and make me feel better at least in the meantime until I'm fully recovered.

This reflects how people reach out when experiencing physical and emotional discomfort, often looking for reassurance or quick solutions to improve wellbeing. Responses vary widely, some offer structured PCT guidance, while others discourage recovery protocols altogether. The competing perspectives within forum discussions influence consumers' decision‐making, shaping whether they engage in PCT, modify their approach or continue AAS use without structured recovery.

Many take a reactive approach, seeking advice only after experiencing negative effects rather than proactively structuring their PCT.

I don't think about PCT and won't if I start feeling like shit then I know I can always find something that helps [online].

This pattern suggests that some individuals prioritise immediate symptom management over long‐term health planning. One AAS consumer acknowledged the debate around PCT protocols:

Now I will agree no PCT is a bad idea, but you certainly don't need 5 drugs for PCT.

Others shared their struggles with inadequate recovery strategies, realising their previous approaches were insufficient:

Last time I came off I got shut down really hard looked into it. It seems like my usual one bottle HCG [human chorionic gonadotropin] and Nolvadex [tamoxifen] is not enough.

This reflects a trial‐and‐error approach, where people who use AAS often adjust their PCT only after experiencing what they perceive to be significant suppression. Forum discussions shape these adjustments, influencing whether consumers adopt more structured recovery plans or remain sceptical of PCT effectiveness.

## 4 Discussion

The aim of this study was to explore online discourses around PCT, including perceived health risks, benefits and barriers, among people who use AAS. A major commonality identified was the way in which AAS consumers utilised forums to assess the risk and necessity of implementing PCT. This assessment is driven by peer conversations around the potential beneficial or adverse outcomes of engaging in PCT. Across these discussions, multiple barriers discouraged PCT uptake. Social influences, such as dominant forum norms and anti‐PCT discourse, shaped consumers' willingness to engage in recovery protocols. Systemic factors, like drug policy, further limited access and created additional risks for those prioritising health. These barriers reinforced a cycle where AAS consumers were either deterred from PCT or forced to navigate it through unofficial, risk‐laden channels. Regardless of the cessation strategy suggested or implemented, AAS consumers sought similar outcomes that reflected healthy hormone levels, reductions in negative psychological effects and maintenance of their ‘*gains*’. However, forum responses displayed some discrepancy regarding the most effective ways to achieve these goals, reflecting differences in expertise and opinion among members, as well as the broader lack of available data on the effectiveness of this endocrine treatment. These ethnopharmacological peer‐discussions often centred on hCG, selective oestrogen receptor modulators, aromatase inhibitors or AAS continuation. Communities debated harm reduction strategies, weighing PCT's necessity against alternatives like low‐dose cruising, balancing anecdotal experiences with scientific literature.

The most discussed AAS‐related symptoms in forum discussions were hypogonadism and general symptoms of fatigue, low energy, mood disturbances and perceived declines in overall health. Participants frequently sought guidance on managing these effects, often linking them to concerns about post‐cycle recovery and long‐term hormonal regulation. Research highlights that AAS‐induced hypogonadism can lead to disruptions in sperm production, testicular atrophy, gynecomastia and, in females, menstrual dysfunction. For men, these challenges are further complicated by the influence of cultural constructions of masculinity, where maintaining physical strength, sexual function and body image are closely tied to identity [14, 17]. Such gendered expectations can intensify anxiety around withdrawal symptoms and prolong reliance on continued AAS use [12]. Current research suggests that in most men, AAS‐induced hypogonadism and sperm production return to normal levels within 3–12 months [52]. While there is evidence for the use of hCG in improving endogenous testosterone production and supporting sperm function in some forms of non‐AAS‐induced hypogonadism [8, 11, 23, 27, 28], there is limited empirical evidence supporting cyclical AAS and PCT use for hormonal recovery [8, 27, 28]. It is also possible that the effect of PCT on endogenous testosterone production is not sustained, and it may even prolong recovery. These findings underscore how information shared in forums can contribute to harm reduction efforts, but also highlight the challenges consumers face in assessing the reliability and validity of advice, which may impact their ability to implement effective harm reduction strategies.

The willingness of AAS consumers to share information within digital spaces reflects the formation of peer‐driven knowledge networks that facilitate harm reduction efforts [9, 10]. These networks can be understood through the lens of ethnopharmacological connoisseurship, whereby situated and contextually relevant knowledge acquired through lived experience becomes a valued and trusted source of health information [41]. This is particularly relevant in contexts such as this where formal healthcare provision is perceived as inadequate. Such networks do not simply replicate biomedical knowledge but adapt and translate it to meet the contextual needs of their members, often embedding it in lived‐experience narratives that increase its credibility and uptake [17]. However, while these spaces are key sites for disseminating information on PCT protocols, the accuracy and reliability of such knowledge remain inconsistent. As with other digital drug communities [53], including those who use AAS generally [36], the processes of knowledge exchange are shaped by both collective problem‐solving and the reinforcement of shared norms. This aligns with broader theories of participatory knowledge production, where authority is distributed across a network rather than concentrated in institutional actors [14, 54, 55]. A key driver of this reliance on peer knowledge is the perceived inadequacy of medical support for AAS consumers [35]. The lack of evidence‐based guidance on AAS cessation and recovery, due to the scarcity of randomised controlled studies in this area, has reinforced the need for alternative spaces where consumers can seek support. In the absence of robust research, guidance is often extrapolated from other medical conditions, which may not always be appropriate. Research has consistently shown that AAS consumers report low confidence in medical professionals' knowledge of AAS [30, 32], and stigma remains a significant barrier to healthcare engagement. Collectively, these factors contribute to a climate in which AAS consumers are forced to navigate misinformation, leading to heightened anxiety about cessation and recovery strategies.

Currently, there is a significant gap in clinical guidelines for evidence‐based PCT and cessation strategies [23, 27]. While peer forums provide a space for information exchange, the lack of evidence‐based protocols has led to the proliferation of conflicting advice, increasing the risk of harm. This knowledge gap reinforces ongoing AAS use as people seek to avoid the negative health effects associated with discontinuation. The challenge, therefore, is not only to improve clinical research on best‐practice cessation strategies but also to develop mechanisms for integrating peer‐driven knowledge translation with emerging medical evidence. By bridging this divide, harm reduction efforts can be strengthened, ensuring that AAS consumers have access to reliable, non‐stigmatising, and actionable information to support their health and wellbeing. Moderation of these forums by experienced people who use AAS in partnership with harm reduction professionals could help bridge this divide. This collaborative approach could help ensure that AAS consumers have access to reliable, non‐stigmatising, and actionable information to support their health and wellbeing. Such an approach mirrors co‐production models in health communication related to AAS, where community members and professionals collaborate to curate, validate and disseminate information [26, 43, 56]. However, addressing these challenges requires more than individual education; structural barriers, particularly those imposed by Australian drug policy, must also be acknowledged [29, 30]. The continued criminalisation of AAS possession and use in Australia [30] exacerbates harm by discouraging people from seeking healthcare support, reinforcing both physiological and psychological barriers to cessation. Fear of legal consequences further limits engagement with harm reduction services, making it essential to prioritise public health approaches over punitive responses. A shift toward education‐driven harm reduction strategies is crucial for promoting long‐term health awareness and risk mitigation.

### 4.1 Limitations

This study has limitations that underscore the need for further research. It is important to acknowledge that the use of forum data likely introduces selection bias, as individuals who post may be those experiencing concerns or adverse effects, which could limit the generalisability of findings to the broader population of AAS consumers. This may affect the external validity of the study. The analysis focused on Australian‐based forums, which provided valuable insights into AAS consumer experiences and PCT perceptions. However, AAS use is a global phenomenon, and recovery and cessation strategies are shaped by diverse legal, cultural and healthcare contexts. Nonetheless, given similarities in AAS use patterns globally [2], it is plausible that many insights from this Australian context may be transferable to other settings. Additionally, more research is needed to explore how masculine identities influence men's engagement with PCT and cessation practices, which may further inform tailored harm reduction and clinical interventions.

## 5 Conclusions

This study highlights the critical role of peer knowledge networks in shaping AAS consumers' understanding of PCT, particularly in the absence of robust, evidence‐based clinical protocols. While forums provide an essential space for harm reduction discussions, the inconsistency of available information underscores the urgent need for evidence‐based cessation strategies. Closing this gap requires dedicated research into robust PCT protocols. Additionally, structural barriers such as stigma and criminalisation further complicate access to cessation and recovery support, reinforcing the need for harm reduction approaches that integrate both medical and community expertise. Without such efforts, AAS consumers will remain caught between incomplete knowledge, persistent health risks and limited pathways to safe discontinuation.

## Author Contributions

**Jonathan Easton:** funding acquisition, data collection, data analysis, manuscript writing and review. **Bonnie Grant:** data analysis, manuscript writing and review. **Geoffrey Spurling:** data analysis, manuscript writing and review. **Raphael Magnolini:** data analysis, manuscript writing and review. **Timothy Piatkowski:** conceptualisation, funding acquisition, ethics approval, data collection, data analysis, supervision, manuscript writing and review.

## Conflicts of Interest

The authors declare no conflicts of interest.

## Data Availability

The data that support the findings of this study are available online. These data were derived from forums available in the public domain.

## References

[1] G. Kanayama and H. G. Pope, Jr. , “History and Epidemiology of Anabolic Androgens in Athletes and Non‐Athletes,” Molecular and Cellular Endocrinology 464 (2018): 4–13. 28245998 10.1016/j.mce.2017.02.039

[2] D. Sagoe , C. S. Andreassen , and S. Pallesen , “The Aetiology and Trajectory of Anabolic‐Androgenic Steroid Use Initiation: A Systematic Review and Synthesis of Qualitative Research,” Substance Abuse Treatment, Prevention, and Policy 9, no. 1 (2014): 1–14. 24984881 10.1186/1747-597X-9-27 PMC4091955

[3] D. Sagoe , H. Molde , C. S. Andreassen , T. Torsheim , and S. Pallesen , “The Global Epidemiology of Anabolic‐Androgenic Steroid Use: A meta‐Analysis and meta‐Regression Analysis,” Annals of Epidemiology 24, no. 5 (2014): 383–398. 24582699 10.1016/j.annepidem.2014.01.009

[4] G. H. Santos and R. Coomber , “The Risk Environment of Anabolic–Androgenic Steroid Users in the UK: Examining Motivations, Practices and Accounts of Use,” International Journal of Drug Policy 40 (2017): 35–43. 27955960 10.1016/j.drugpo.2016.11.005

[5] M. Salinas , W. Floodgate , and R. Ralphs , “Polydrug Use and Polydrug Markets Amongst Image and Performance Enhancing Drug Users: Implications for Harm Reduction Interventions and Drug Policy,” International Journal on Drug Policy 67 (2019): 43–51. 30884355 10.1016/j.drugpo.2019.01.019

[6] S. Heard and L. Maher , “Australian Needle Syringe Program Survey National Data,” 2024.

[7] A. Sharma , B. Grant , H. Islam , A. Kapoor , A. Pradeep , and C. N. Jayasena , “Common Symptoms Associated With Usage and Cessation of Anabolic Androgenic Steroids in Men,” Best Practice & Research. Clinical Endocrinology & Metabolism 36, no. 5 (2022): 101691. 35999138 10.1016/j.beem.2022.101691

[8] B. Grant , J. Campbell , A. Pradeep , et al., “Factors Predicting Normalization of Reproductive Hormones After Cessation of Anabolic‐Androgenic Steroids in Men: A Single Center Retrospective Study,” European Journal of Endocrinology 189, no. 6 (2023): 601–610. 38102386 10.1093/ejendo/lvad164

[9] H. Lamb , M. Dunn , I. A. Havnes , and T. Piatkowski , ““I Go Back to It Every f** King Time”: The Normalization of Problematic Trenbolone Use in Online Anabolic‐Androgenic Steroid Communities,” Addiction Research & Theory 33 (2024): 1–7.40059906

[10] B. Tighe , M. Dunn , F. H. McKay , and T. Piatkowski , “Information Sought, Information Shared: Exploring Performance and Image Enhancing Drug User‐Facilitated Harm Reduction Information in Online Forums,” Harm Reduction Journal 14 (2017): 48. 28732534 10.1186/s12954-017-0176-8 PMC5521146

[11] M. A. Christou , P. A. Christou , G. Markozannes , A. Tsatsoulis , G. Mastorakos , and S. Tigas , “Effects of Anabolic Androgenic Steroids on the Reproductive System of Athletes and Recreational Users: A Systematic Review and Meta‐Analysis,” Sports Medicine 47, no. 9 (2017): 1869–1883. 28258581 10.1007/s40279-017-0709-z

[12] S. Griffiths , R. Henshaw , F. H. McKay , and M. Dunn , “Post‐Cycle Therapy for Performance and Image Enhancing Drug Users: A Qualitative Investigation,” Performance Enhancement & Health 5, no. 3 (2017): 103–107.

[13] G. Kanayama , J. I. Hudson , and H. G. Pope, Jr. , “Long‐Term Psychiatric and Medical Consequences of Anabolic–Androgenic Steroid Abuse: A Looming Public Health Concern?,” Drug and Alcohol Dependence 98, no. 1–2 (2008): 1–12. 18599224 10.1016/j.drugalcdep.2008.05.004 PMC2646607

[14] G. Nourse , D. Moore , and S. Fraser , “Who Veridicts Health? Health Professional Discourses on Performance and Image‐Enhancing Drugs (PIEDs), health, and Masculinity,” Contemporary Drug Problems 51 (2024): 00914509241288253.

[15] E. Hearne , A. Atkinson , I. Boardley , J. McVeigh , and M. C. Van Hout , “‘Sustaining Masculinity’: A Scoping Review of Anabolic Androgenic Steroid Use by Older Males,” Drugs Education, Prevention and Policy 31, no. 1 (2022): 27–53.

[16] T. M. Piatkowski , K. M. White , L. M. Hides , and P. L. Obst , “Australia's Adonis: Understanding What Motivates Young Men's Lifestyle Choices for Enhancing Their Appearance,” Australian Psychologist 55, no. 2 (2020): 156–168.

[17] G. Nourse , S. Fraser , and D. Moore , “Masculine Enhancement as Health or Pathology: Gender and Optimisation Discourses in Health Promotion Materials on Performance and Image‐Enhancing Drugs (PIEDs),” Health Sociology Review 33 (2024): 1–305. 38286142 10.1080/14461242.2023.2297046

[18] A. B. Parkinson and N. A. Evans , “Anabolic Androgenic Steroids: A Survey of 500 Users,” Medicine and Science in Sports and Exercise 38, no. 4 (2006): 644–651. 16679978 10.1249/01.mss.0000210194.56834.5d

[19] D. L. Smit , O. de Hon , B. J. Venhuis , M. den Heijer , and W. de Ronde , “Baseline Characteristics of the HAARLEM Study: 100 Male Amateur Athletes Using Anabolic Androgenic Steroids,” Scandinavian Journal of Medicine & Science in Sports 30, no. 3 (2020): 531–539. 31663164 10.1111/sms.13592

[20] S. Karavolos , M. Reynolds , N. Panagiotopoulou , K. McEleny , M. Scally , and R. Quinton , “Male Central Hypogonadism Secondary to Exogenous Androgens: A Review of the Drugs and Protocols Highlighted by the Online Community of Users for Prevention and/or Mitigation of Adverse Effects,” Clinical Endocrinology 82, no. 5 (2015): 624–632. 25333666 10.1111/cen.12641

[21] W. de Ronde and D. L. Smit , “Anabolic Androgenic Steroid Abuse in Young Males,” Endocrine Connections 9, no. 4 (2020): R102–R111. 32229704 10.1530/EC-19-0557 PMC7219134

[22] M. Rochoy , A. Danel , E. Chazard , S. Gautier , and C. Berkhout , “Doping With Aromatase Inhibitors and Oestrogen Receptor Modulators in Steroid Users: Analysis of a Forum to Identify Dosages, Durations and Adverse Drug Reactions,” Thérapie 77 (2022): 683–691. 35660259 10.1016/j.therap.2022.03.004

[23] C. N. Jayasena , R. A. Anderson , S. Llahana , et al., “Society for Endocrinology Guidelines for Testosterone Replacement Therapy in Male Hypogonadism,” Clinical Endocrinology 96, no. 2 (2022): 200–219. 34811785 10.1111/cen.14633

[24] M. Huijben , M. T. W. Lock , V. F. de Kemp , L. M. de Kort , and H. Van Breda , “Clomiphene Citrate for Men With Hypogonadism: A Systematic Review and meta‐Analysis,” Andrology 10, no. 3 (2022): 451–469. 34933414 10.1111/andr.13146

[25] E. P. Wenker , J. M. Dupree , G. M. Langille , et al., “The Use of HCG‐Based Combination Therapy for Recovery of Spermatogenesis After Testosterone Use,” Journal of Sexual Medicine 12, no. 6 (2015): 1334–1337. 25904023 10.1111/jsm.12890

[26] T. Piatkowski , R. Coomber , C. Francis , et al., “The World's First Anabolic‐Androgenic Steroid Testing Trial: A Two‐Phase Pilot Combining Chemical Analysis, Results Dissemination and Community Feedback,” Addiction 120, no. 7 (2025): 1366–1377. 39911049 10.1111/add.70009 PMC12128565

[27] B. Grant , J. Kean , N. Vali , et al., “The Use of Post‐Cycle Therapy Is Associated With Reduced Withdrawal Symptoms From Anabolic‐Androgenic Steroid Use: A Survey of 470 Men,” Substance Abuse Treatment, Prevention, and Policy 18, no. 1 (2023): 66. 37951896 10.1186/s13011-023-00573-8 PMC10640727

[28] B. Grant , J. Campbell , A. Pradeep , et al., “OR25‐03 Self‐Administration of Post‐Cycle Therapy Is Associated With Increased Probability of Subsequent Normalisation of Reproductive Hormones Following Anabolic‐Androgenic Steroid Cessation in Men,” Journal of the Endocrine Society 7, no. Supplement_1 (2023): bvad114.

[29] T. Piatkowski , L. Ayurzana , M. King , L. Hattingh , and S. McMillan , “Community Pharmacy's Role in Dispensing Androgens and Supporting Harm Reduction Amid Current Policy Dilemmas,” Substance Abuse Treatment, Prevention, and Policy 20, no. 1 (2025): 1–12. 39827172 10.1186/s13011-025-00636-y PMC11748596

[30] T. Piatkowski , N. Gibbs , and M. Dunn , “Beyond the Law: Exploring the Impact of Criminalising Anabolic–Androgenic Steroid Use on Help‐Seeking and Health Outcomes in Australia,” Journal of Criminology 57, no. 1 (2024): 62–82.

[31] T. Piatkowski , S. Benn , L. Ayurzana , M. King , S. McMillan , and L. Hattingh , “Exploring the Role of Community Pharmacies as a Harm Reduction Environment for Anabolic–Androgenic Steroid Consumers: Triangulating the Perspectives of Consumers and Pharmacists,” Harm Reduction Journal 21, no. 1 (2024): 59. 38481218 10.1186/s12954-024-00972-5 PMC10935940

[32] A. Richardson , J. Kean , L. Fleming , J. I. Hudson , G. Kanayama , and H. G. Pope, Jr. , “Attitudes of Anabolic Steroid Users and Non‐Users Towards General Practitioners in the United Kingdom,” Performance Enhancement & Health 12, no. 4 (2024): 100304.

[33] L. Cox , T. Piatkowski , and J. McVeigh , ““I Would Never Go to the Doctor and Speak About Steroids”: Anabolic Androgenic Steroids, Stigma and Harm,” Drugs: Education, Prevention and Policy (2024): 1–13.40206199

[34] J. McVeigh and G. Bates , “Stigma and the Use of Anabolic Androgenic Steroids by Men in the United Kingdom,” in Drugs, Identity and Stigma (Springer, 2022), 121–146.

[35] S. Fraser , R. Fomiatti , D. Moore , K. Seear , and C. Aitken , “Is Another Relationship Possible? Connoisseurship and the Doctor–Patient Relationship for Men Who Consume Performance and Image‐Enhancing Drugs,” Social Science & Medicine 246 (2020): 112720. 31972378 10.1016/j.socscimed.2019.112720

[36] J. Andreasson and A. Henning , Online Doping: The Digital Ecosystem and Cyborgification of Drug Cultures (Springer Nature, 2023).

[37] A. Henning and J. Andreasson , ““Yay, Another Lady Starting a Log!”: Women's Fitness Doping and the Gendered Space of an Online Doping Forum,” Communication & Sport 9, no. 6 (2021): 988–1007.

[38] M. Hawk , R. W. Coulter , J. E. Egan , et al., “Harm Reduction Principles for Healthcare Settings,” Harm Reduction Journal 14 (2017): 1–9. 29065896 10.1186/s12954-017-0196-4 PMC5655864

[39] L. F. Monaghan , “Vocabularies of Motive for Illicit Steroid Use Among Bodybuilders,” Social Science & Medicine 55, no. 5 (2002): 695–708. 12190264 10.1016/s0277-9536(01)00195-2

[40] M. Underwood , “From ‘Bro, Do You Even Lift?’to ‘Bro, Do You Even Science?’: How the Relationship Between Science and Broscience Can Inform the Development of Allied Image and Performance Enhancing Drug Harm Reduction,” Performance Enhancement & Health (2024): 100291.

[41] T. Piatkowski , N. Gibbs , D. Neumann , and M. Dunn , “Consuming ‘God Juice’: Using ‘Ethnopharmacological‐Connoisseurship’ to Situate Trenbolone Use and Knowledge Among Image and Performance Enhancing Drug Communities,” Drug and Alcohol Review 44, no. 1 (2024): 298–309. 39433468 10.1111/dar.13965

[42] V. Champion and C. S. Skinner , “The Health Belief Model,” in Health Behavior and Health Education, ed. K. Glanz , B. Rimer , and K. Viswanath , vol. 4 (Jossey‐Bass, 2008), 45–65.

[43] T. Piatkowski , R. Coomber , C. Francis , et al., “Anabolic‐Androgenic Steroid Testing as a Tool for Consumer Engagement and Harm Reduction: A Sequential Explanatory Mixed‐Method Study,” Harm Reduction Journal 22, no. 1 (2025): 114. 40616139 10.1186/s12954-025-01270-4 PMC12231991

[44] C. Hewson and T. Buchanan , Ethics Guidelines for Internet‐Mediated Research (British Psychological Society, 2013).

[45] T. M. Piatkowski , K. M. White , L. M. Hides , and P. L. Obst , “The Impact of Social Media on Self‐Evaluations of Men Striving for a Muscular Ideal,” Journal of Community Psychology 49, no. 2 (2021): 725–736. 33295649 10.1002/jcop.22489

[46] N. Gibbs and T. Piatkowski , “The Liver King Lie: Misrepresentation, Justification, and Public Health Implications,” International Journal on Drug Policy 114 (2023): 103979. 36841216 10.1016/j.drugpo.2023.103979

[47] M. J. Gill , T. Piatkowski , and M. Dunn , “A Netnographic Study of Anabolic‐Androgenic Steroid Initiation Videos on YouTube,” Drug and Alcohol Review 44, no. 1 (2025): 310–323. 39462946 10.1111/dar.13969

[48] R. Kozinets , Netnography: Redefined (Sage, 2015).

[49] J. Neale , “Iterative Categorization (IC): A Systematic Technique for Analysing Qualitative Data,” Addiction 111, no. 6 (2016): 1096–1106. 26806155 10.1111/add.13314 PMC5069594

[50] J. Neale , “Iterative categorisation (IC) (part 2): interpreting qualitative data,” Addiction 116, no. 3 (2021): 668–676. 32926762 10.1111/add.15259

[51] A. J. Fletcher , “Applying Critical Realism in Qualitative Research: Methodology Meets Method,” International Journal of Social Research Methodology 20, no. 2 (2017): 181–194.

[52] D. Smit , M. Buijs , O. De Hon , M. Den Heijer , and W. De Ronde , “Disruption and Recovery of Testicular Function During and After Androgen Abuse: The HAARLEM Study,” Human Reproduction 36, no. 4 (2021): 880–890. 33550376 10.1093/humrep/deaa366

[53] A. Maddox , M. J. Barratt , M. Allen , and S. Lenton , “Constructive Activism in the Dark Web: Cryptomarkets and Illicit Drugs in the Digital ‘Demimonde’,” Information, Communication & Society 19, no. 1 (2016): 111–126.

[54] S. Adams , T. Rhodes , and K. Lancaster , “New Directions for Participatory Modelling in Health: Redistributing Expertise in Relation to Localised Matters of Concern,” Global Public Health 17, no. 9 (2022): 1827–1841. 34775919 10.1080/17441692.2021.1998575

[55] L. M. Boucher , Z. Marshall , A. Martin , et al., “Expanding Conceptualizations of Harm Reduction: Results From a Qualitative Community‐Based Participatory Research Study With People Who Inject Drugs,” Harm Reduction Journal 14, no. 1 (2017): 1–18. 28494774 10.1186/s12954-017-0145-2 PMC5427533

[56] T. Piatkowski , I. Volpe , R. Brien , et al., “Development, Dissemination and Community Response Towards the First Community Notice Regarding Misrepresented Illicit Anabolic‐Androgenic Steroids in Circulation in Australia,” Drug and Alcohol Review 44, no. 3 (2025): 735–741. 39930659 10.1111/dar.14015 PMC11886500

